# Exploration of Spatio-Temporal Characteristics of Carbon Emissions from Energy Consumption and Their Driving Factors: A Case Analysis of the Yangtze River Delta, China

**DOI:** 10.3390/ijerph19159483

**Published:** 2022-08-02

**Authors:** Weiwu Wang, Huan Chen, Lizhong Wang, Xinyu Li, Danyi Mao, Shan Wang

**Affiliations:** 1 College of Civil Engineering and Architecture, Zhejiang University, Hangzhou 310058, China; chenhuan01@zju.edu.cn (H.C.); lizhongwang@zju.edu.cn (L.W.); lixinyu1001@zju.edu.cn (X.L.); maodanyi0122@zju.edu.cn (D.M.); wangshan33@zju.edu.cn (S.W.); 2 China of Institute of Urbanization, Zhejiang University, Hangzhou 310058, China; 3 Center for Balance Architecture, Zhejiang University, Hangzhou 310058, China

**Keywords:** carbon emissions from energy consumption, decoupling elasticity, spatio-temporal characteristics, improved LMDI model, k-means clustering, map visualization

## Abstract

For the Yangtze River Delta (YRD) region of China, exploring the spatio-temporal characteristics of carbon emissions from energy consumption (CEECs) and their influencing factors is crucial to achieving carbon peaking and carbon neutrality as soon as possible. In this study, an improved LMDI decomposition model based on the Tapio model and Kaya’s equation was proposed. Combined with the improved LMDI and k-means cluster analysis methods, the energy structure, energy intensity, unit industrial output value and population size were selected as the driving factors, and the contribution of each driving factor to the CEECs of prefecture-level cities was quantitatively analyzed. Our study found that: (1) By 2020, the total amount of CEECs in the 26 prefecture-level cities in the YRD will stabilize, while their intensity has shown a downward trend in recent years. (2) The decoupling relationship between CEECs and economic development generally showed a trend from negative decoupling to decoupling. The dominant factor in decoupling was generally the shift of *DEL* values towards urbanization rate and energy intensity and the open utilization of energy technologies. (3) From 2000 to 2010, the dominant factors affecting CEECs in 26 cities were energy intensity and energy structure, followed by industrial output value and urbanization rate. In general, the promotion effect of economic development on carbon emissions in the YRD region was greater than the inhibitory effect. After 2010, the restrictive effect of various factors on CEECs increased significantly, among which the role of gross industrial output was crucial. The research results can provide a scientific policy basis for the subsequent spatial management and control of carbon emission reduction and carbon neutrality in the YRD region at a finer scale.

## 1. Introduction

In recent years, Chinese local governments, relevant departments and critical industries have worked together to reduce carbon emissions and have set emission reduction targets in their mid- and long-term economic development plans [1]. China has set a goal of peaking carbon emissions by 2030 and is striving to achieve carbon neutrality by 2060 [2]. However, China’s current emission reduction plan targets are mostly set at the national and provincial levels. During the implementation of the actual emission target, due to the huge differences in regional economic development, industrial structure and energy structure, the different dynamic factors affecting carbon emissions are also complex and changeable. Therefore, analyzing the spatio-temporal characteristics, decoupling relationships and driving factors of carbon emissions in critical regions or cities in China is of great practical significance for effectively formulating carbon emission mitigation targets for regions or cities [3].

As the most active urban agglomeration in China, the YRD region is not only the region with the fastest economic growth, the largest economic aggregate and the greatest economic potential in China, but also a critical control area for achieving carbon peaking and carbon neutrality goals [4]. The YRD urban agglomeration is an essential industrial intersection between the “Belt and Road Initiative” and the Yangtze River economic belt. It is also a demonstration area for high-quality integrated development in China. In 2020, the total GDP will account for about 15% of the country’s total [5], the urban population will account for about 17% of the country’s total urban population and the total carbon emissions will account for about 16% of the country’s total carbon emissions [6]. It is a crucial control area for achieving carbon peaking and carbon neutrality [7], and it is also an essential leader in China’s achieving the dual-carbon goal.

The decomposition analysis of the CEECs’ driving factors is the basis for achieving the regional carbon emission target. In recent years, many studies have explored the path of effective emission reduction and low-carbon green economic development by analyzing the decoupling relationship between economic growth, energy consumption and CO_2_ emissions. Currently, decomposition analysis research mainly focuses on the CEECs from various industries such as the transportation industry, textile industry, manufacturing industry, non-metallic minerals and residential CEECs. The research on the decomposition analysis of decoupling relationships mainly focuses on the CEECs in various industries such as transportation [8], textile [9], manufacturing [10], non-metallic minerals [11] and real estate [12]. Ma et al. [13] used the LMDI decomposition analysis method to study seven energy consumption sectors in China and found that eliminating excess capacity and promoting structural transformation has become the only way for China to reduce emissions. Xu et al. [14] analyzed the decomposition of CEECs based on the two dimensions of China’s various periods and industries and studied the main factors that promote and suppress CEECs in China at different stages. Yang et al. [15] emphasized that economic energy consumption is the biggest driver of carbon emission growth in China and pointed out that introducing electricity import measures can buffer the impact of the carbon emission intensity of annual energy consumption. Liu et al. [16] classified the emission reduction of each city from the perspective of periods and groups based on the decomposition analysis of CEECs at the provincial level in China. As the world’s second-largest economy and a developing country with a large population base, high degree of industrialization and high dependence on coal, China has a long way to go to reduce emissions. Therefore, understanding the various characteristics of CEECs and the driving factors of CEECs has always been the direction for China’s cities to follow when exploring low-carbon economic development and ecological construction.

According to the environmental Kuznets curve (EKC) hypothesis, there is an inverted U-shaped relationship between environmental pressure and economic growth. That is, in the early stage of economic development, environmental quality deteriorates with economic growth. Environmental quality gradually improves when economic development reaches a certain level [17]. The “decoupling” theory is a basic theory proposed by the Organization for Economic Cooperation and Development to describe the connection between economic growth and resource consumption or environmental pollution (Paris: OECD, 2002). Decoupling of carbon emissions, which, in essence, is to measure whether economic growth is at the cost of resource consumption and environmental damage, can be used to describe the relationship between changes in CO_2_ emissions and economic growth. When economic growth is achieved, if the growth rate of CO_2_ emissions is negative or lower than the economic growth rate, it can be regarded as decoupling. That is, based on economic growth, energy consumption is gradually reduced [18]. Zhang [19] introduced the decoupling index in energy and environment research in 2000, and Freitas et al. [20] used this method to explore the decoupling between economic activity and CEECs in Brazil from 2004 to 2009. In addition, in 2005, Tapio [21] proposed a theoretical framework for decoupling, which defined the difference between “decoupling”, “connection” and “negative decoupling” and then divided them into “weak”, “strong”, “extended” and “decline”. Some existing studies used decomposition analysis models and clustering methods to study the decoupling relationship between carbon emissions and China’s economic development. Zhang et al. [22] used the LMDI method to decompose the decoupling index between economic growth and energy consumption in China from 1991 to 2012. The results showed that economic activity negatively impacted the decoupling. Chang et al. [23] used a new regional classification framework combining factor analysis and Ward clustering to divide 30 provinces in China into four regions and investigated the differences in the impacts of population size, per capita GDP, energy structure and energy intensity on CO_2_ emissions. In summary, the existing literature has achieved important results regarding the decoupling relationship between economic development and CEECs. As an essential demonstration area for economic development and CO_2_ emission control in China, the YRD region is committed to promoting China’s deep decarbonization and achieving high-quality economic development.

## 2. Literature Review

### 2.1. Empirical Study on Energy Carbon Emissions in the YRD Region

In recent years, some studies have gradually been carried out on low-carbon emission reduction in the YRD region. By formulating a green economy model, the World Resources Institute (WRI) predicted that the YRD region will reach a carbon peak in 2024 under the background of a green economy, with a peak value of about 1.793 billion tons of carbon dioxide equivalent and the peak forecast will be reduced by 90 million tons of carbon dioxide equivalent, which is two years earlier than the current background and policy deduction to achieve the carbon peak, and this benefit can lead the national carbon reduction action (WRI, China, 2021). At the same time, many researchers are also actively committed to research on emission reduction in the YRD region. Song et al. [24] calculated the annual variation of energy consumption in the YRD region from 1995 to 2010 and proposed ways to control carbon emissions in the YRD region against the background of sustained economic growth. Gong et al. [25] used the STIRPAT model to quantitatively analyze the relationship between CO_2_ emissions in the YRD and influencing factors such as population, per capita GDP, foreign direct investment and technological progress. Pei et al. [26] investigated the carbon footprint from fossil energy consumption and the decoupling relationship between carbon footprint pressure and economic growth in the YRD combined with land resource constraints. They combined gap analysis and social network analysis. Shen et al. [27] found that the YRD highlights the strong collaborative development ability and driving ability of developed cities, thus generating the greatest potential to reduce CO_2_ emissions in the short and medium terms. In summary, it can be seen that the research on carbon emissions in the YRD region has become a typical research model relating to low-carbon emissions in China’s urban agglomeration. However, the influencing factors of the decoupling relationship between all carbon emissions and economic development in the YRD region and their temporal and spatial distribution still need further research. Therefore, an in-depth study of energy consumption and carbon emissions in the YRD region to provide policymakers with information to achieve carbon emission reduction targets is crucial for helping China’s economically developed regions achieve future emission reduction targets.

### 2.2. Methods for Identifying Drivers of Carbon Emissions

At present, the application of the LMDI method in the field of energy and environment can be mainly divided into three directions: (1) The LMDI method is directly used to explore the influencing factors of carbon emissions. For example, Quan et al. used the LMDI method to decompose the carbon emission factors of China’s logistics industry from 2000 to 2016 into five dimensions, carbon emission factors, energy intensity, energy structure, economic development level and population size, and the carbon emission contribution rates were analyzed separately [28]. (2) As an evaluation model, LMDI is combined with other models to decompose and analyze the data obtained by other models and conduct in-depth evaluation and analysis. The current application in the field of carbon emission research is mainly combined with the decoupling model to further study the low-carbon development of cities in a quantitative manner. For example, Wang et al. [29] combined the Tapio decoupling index and the LMDI model to decompose the factors affecting energy consumption and carbon emissions and put forward the focus of promoting green and low-carbon development and the transformation of Qinghai Province. (3) As a previous decomposition model, the relationship of influencing factors obtained by decomposition is mainly used for subsequent analysis, such as peak prediction, situation simulation, etc. For example, Zhang et al. [30] combined the scenario analysis method with the Monte Carlo prediction method, using LMDI to decompose the driving factors of China’s total water consumption to predict the trend of China’s water consumption change before 2030 and then judge the peak time point and occurrence of water consumption. Gu et al. [31] combined LMDI with a system dynamics model (SD method) to quantify and estimate emission reduction potential in Shanghai, China. In addition, the application of LMDI is not limited to the traditional energy field but also includes economic cost estimation and related patent research. For example, Zhang et al. [32] investigated the relevant biogas user data in 19 villages in China in 2015, quantified the gap between the theoretical cost and actual cost of CO_2_ emission reduction per unit and analyzed the main factors affecting the cost with the help of the LMDI model.

Our main research purpose is to go deep into 26 prefecture-level cities in the YRD region to reveal the spatio-temporal changes in the decoupling relationship between carbon emissions and economic development. At the same time, LMDI is introduced as an evaluation model, the data obtained by the decoupling model are decomposed and analyzed and the decoupling elasticity of urbanization rate, energy intensity, unit industrial output value and energy structure is deeply evaluated. Based on previous research, the k-means clustering analysis method is further used to cluster the results, and the leading factors of carbon emissions in cities at various levels in the YRD region are summarized. Specifically, this study aims to reveal the spatio-temporal characteristics and influencing factors of CEECs in prefecture-level cities in the YRD region, the spatio-temporal characteristics and decoupling factors of CEECs from 2000 to 2020 and the contribution rate of each driving factor to CEECs. On the one hand, this will help the YRD region to decompose the national emission reduction targets into local-level cities and, on the other hand, formulate effective emission reduction measures according to local conditions to achieve the goal of pilot testing in the YRD region.

The remainder of this paper is organized as follows. In the next section, we present an overview of the study area. In Section 4, we offer a flowchart of the method used in this study and the data sources. Section 5 reports the main results. Section 6 presents the conclusions and policy implications.

## 3. Study Area

**Figure 1:**
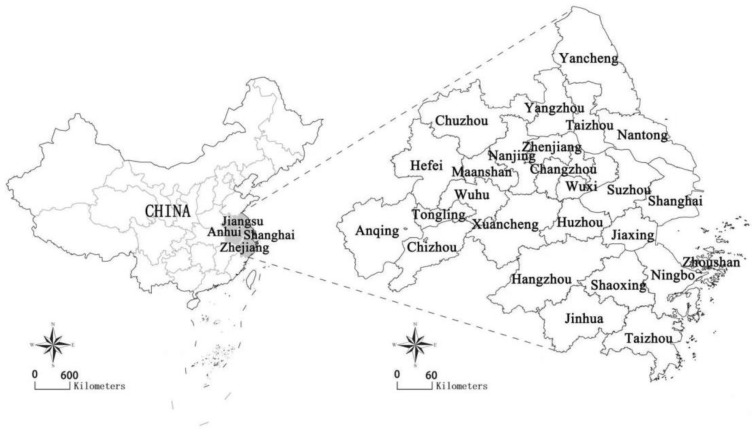
26 prefecture-level cities in the YRD.

This paper selected the YRD region as the research object. The research area covers 26 cities, including Shanghai, Nanjing, Wuxi, Changzhou, Suzhou, Nantong, Yancheng, Yangzhou, Zhenjiang, Taizhou, Hangzhou, Ningbo, Jiaxing, Huzhou, Shaoxing, Jinhua, Zhoushan, Taizhou, Hefei, Wuhu, Ma’anshan, Tongling, Anqing, Chuzhou, Chizhou and Xuancheng (Figure 1).

The YRD region is located in East China, covering 211,700 square kilometers, accounting for about 2.2% of China’s land area. In 2019, the GDP of the YRD region was about CNY 20 trillion, accounting for about 20% of China’s GDP. At the end of the year, the resident population was about 160 million, representing more than one-tenth of the country’s resident population. As an important functional area leading China’s regional economic development, the YRD region has a vast economic hinterland with a modern transportation network and advantageous industrial clusters centered on electronics, automobiles, modern finance and other industries, as well as rich scientific and educational resources.

## 4. Methods

### 4.1. Data Sources

This paper includes three parts of research data. The first is urban statistical data from the 2006–2019 “China Urban Statistical Yearbook”, “China Urban Construction Statistical Yearbook”, “China Energy Statistical Yearbook”, “Jiangsu Statistical Yearbook”, “Zhejiang Statistical Yearbook” and “Anhui Statistical Yearbook”, as well as statistical yearbooks and social development statistical reports of 26 cities, including the main energy consumption statistics of the above-scale industries in the local-level cities from 2000 to 2020, industrial output value, urbanization rate, comprehensive energy consumption and other indicators. Except for a few city data from corporate websites, statistical data were provided by CNKI China Social and Economic Big Data Research Platform, and the link is https://data.cnki.net/HomeNew/index (accessed on 31 January 2022).

### 4.2. Methodology

**Figure 2:**
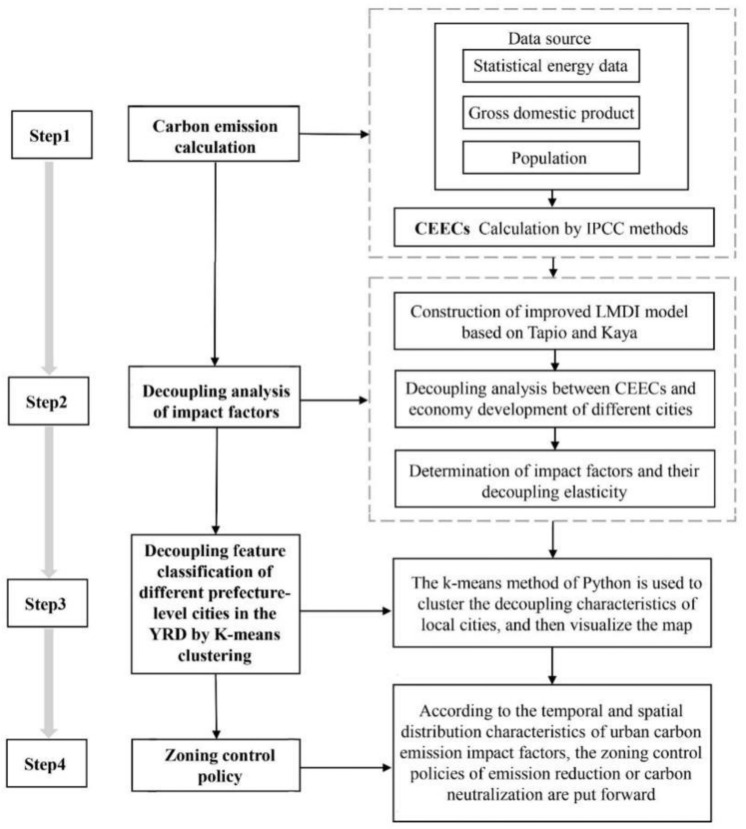
Flowchart of the methodology used in this study.

The specific analysis process is shown in Figure 2. It mainly included using IPCC urban carbon emission calculation, the improved LMDI model based on the Kaya equation and Tapio to analyze each factor’s decoupling elasticity and the k-means cluster analysis and map classification to visualize carbon emission characteristics and carbon emission impact factors for regional management and control analysis.

#### 4.2.1. CEEC Model

Referring to the method proposed by Ang [33], the CEEC calculation formula is as follows:

(1) $$C^{mn}=\sum_{i}E_{i}^{mn}\times ei\times pi\times 44/12$$

where m represents the city m of the 26 cities in the YRD, n represents the node year n from 2000 to 2020, $C^{mn}$ represents the CEEC of the mth city in the node year n, i represents the ith type of fossil energy, $E_{i}^{mn}$ represents the consumption of the *i*th fossil energy in the mth city in the node year n, *ei* represents the conversion coefficient of standard coal to the *i*th energy, which is taken from the General Principles of Comprehensive Energy Consumption Calculation (GB/T2589- 2020), and pi represents the carbon emission factor, which is taken from the IPCC reference value. In the equation, 44/12 represents the molecular weight ratio of carbon dioxide to carbon. The standard coal reduction coefficients and carbon emission coefficients for 21 fossil energy sources are shown in Table 1.

#### 4.2.2. The Improved LMDI Model Based on Kaya Equation and Tapio

In recent years, many scholars have used the concept of decoupling and its indicators to reflect the relationship between economic growth and CO_2_ emissions and have used decoupling elasticity as the main tool to measure the low-carbon status of various regions [14]. In our study, the Tapio model was selected to calculate the decoupling elasticity value, and 26 prefecture-level cities in the YRD were classified and analyzed.

The formula for calculating the elasticity coefficient of the Tapio model is as follows:

(2) $$D^{m}=\frac{\Delta C_{t2-t1}^{m}/C_{t1}^{m}}{\Delta GIO_{t2-t1}^{m}/GIO_{t1}^{m}}$$

where m represents the city m of the 26 cities in YRD, $D^{m}$ represents the decoupling elasticity coefficient of the city m between the two node years t1 and t2 and Δ$C^{m}$ and Δ$GIO^{m}$ represent the changes in carbon emissions and gross industrial output value of the city m in node year t2 relative to node year t1, respectively. Parameters t1 and t2 represent the base node year and the end node year in the study from 2000 to 2020, respectively.

According to the magnitude of the decoupling elasticity and the positive and negative conditions of Δ*C* and Δ*GIO*, Tapio divides the decoupling state into eight decoupling states, as shown in Table 2 [21].

The Kaya identity proposed by Kaya [34] can be used to investigate the influencing factors of changes in greenhouse gas emissions at the national or regional level. The role of this identity is to describe the relationship between social, economic, energy, carbon emissions and other factors with simple mathematical relationships from an overall, macroscopic perspective.

We selected energy structure, energy intensity, the ratio of industrial output value to urbanization rate and urbanization rate as the analysis objects to construct the energy consumption carbon emissions of typical cities in the YRD. The Kaya identity can be expressed as follows:

(3) $$C=\frac{C}{TOE}\times \frac{TOE}{GIO}\times \frac{GIO}{POP}\times POP\;=ES\times EI\times EL\times P$$

where *C* represents the carbon emissions from energy consumption, *TOE* represents the total energy consumption, *GIO* represents the gross industrial production and *POP* represents the urbanization rate. *ES*, *EI*, *EL* and *P* on the right-hand side of the equation represent energy structure, energy intensity, the industrial output value of “unit urbanization rate” and urbanization level, respectively.

Referring to the previous study, the formula for the LMDI method is as follows:

(4) $$\Delta C=C_{t2}-C_{t1}\;=\Delta C_{ES}+\Delta C_{EI}+\Delta C_{EL}+\Delta C_{P}$$

where Δ*C_ES_* represents the energy structure effect, Δ*C_EI_* represents the energy intensity effect and Δ*C_EL_* represents the unit industrial output value effect; Δ*C_P_* represents the population scale effect.

Combined with the above decoupling model, the logarithmic decomposition model of the LMDI method can be expressed as follows:

(5) $$\begin{array}{cc} D & =\frac{GIO_{t1}^{m}}{\Delta GIO^{m}\times C_{t1}^{m}}\times \Delta C_{t2-t1}^{m}\\ & =\frac{GIO_{t1}^{m}}{\Delta GIO^{m}\times C_{t1}^{m}}\times \left(\Delta C_{ES}^{m}+\Delta C_{EI}^{m}+\Delta C_{EL}^{m}+\Delta C_{P}^{m}\right) \end{array}$$

(6) $$DES^{m}=\frac{GIO_{t1}^{m}}{\Delta GIO^{m}\times C_{t1}^{m}}\times \sum_{i^{m}}\frac{\Delta C_{t2-t1}^{m}}{lnC_{t2}^{m}-lnC_{t1}^{m}}\times ln\frac{ES_{t2}^{m}}{ES_{t1}^{m}}$$

(7) $$DEI^{m}=\frac{GIO_{t1}^{m}}{\Delta GIO^{m}\times C_{t1}^{m}}\times \sum_{i^{m}}\frac{\Delta C_{t2-t1}^{m}}{lnC_{t2}^{m}-lnC_{t1}^{m}}\times ln\frac{EI_{t2}^{m}}{EI_{t1}^{m}}$$

(8) $$DEL^{m}=\frac{GIO_{t1}^{m}}{\Delta GIO^{m}\times C_{t1}^{m}}\times \sum_{i^{m}}\frac{\Delta C_{t2-t1}^{m}}{lnC_{t2}^{m}-lnC_{t1}^{m}}\times ln\frac{EL_{t2}^{m}}{EL_{t1}^{m}}$$

(9) $$DP^{m}=\frac{GIO_{t1}^{m}}{\Delta GIO^{m}\times C_{t1}^{m}}\times \sum_{i^{m}}\frac{\Delta C_{t2-t1}^{m}}{lnC_{t2}^{m}-lnC_{t1}^{m}}\times ln\frac{P_{t2}^{m}}{P_{t1}^{m}}$$

where m represents the city m of the 26 cities in the YRD, $DES^{m}$ represents the decoupling elasticity of energy structure of city *m*, $DEI^{m}$ represents the decoupling elasticity of energy intensity of city *m*, $DEL^{m}$ represents the decoupling elasticity of unit industrial output value of city *m*, $DP^{m}$ represents the decoupling elasticity of population size of city *m* and parameters t1 and t2 represent the base node year and the end node year in the study from 2000 to 2020, respectively.

#### 4.2.3. K-Means Method

After decoupling the CEECs of each prefecture-level city, the next important task was to fully reveal the spatial and temporal differences in the driving factors of CEECs in each prefecture-level city. The main idea of k-means clustering method is to use k centroids to cluster multiple discrete data points. The essence is to group the points with higher similarity into a group and separate the points with lower similarity. The method converges to the optimal solution by continuously updating the position of the group centroid. In our study, Python 3.7 was combined with the elbow method described above to determine the optimal number of sets. Then, the k-means method for clustering was used to set the optimal number of groups found by the elbow method as the number of clusters to obtain the final specific number of groups.

## 5. Results and Analysis

### 5.1. Variation Analysis of Total CEECs and CEEC Intensity in the YRD

**Figure 3:**
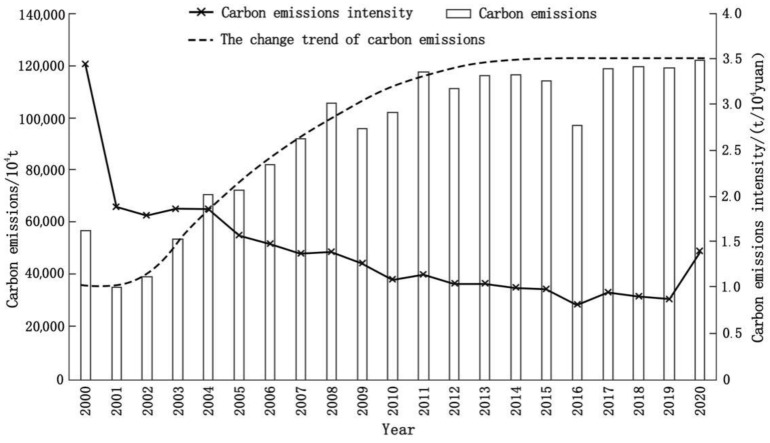
Status of energy carbon emissions in the YRD from 2000 to 2020.

As shown in Figure 3, there were differences in the total amount of CEECs and the intensity of CEECs in the YRD region from 2000 to 2020. The total amount of CEECs increased from 569.75 million tons to 1221.28 million tons, with an average annual growth rate of about 3.89%, and CEEC was basically stable with small fluctuations after 2011. Between 2000 and 2020, the CEEC intensity in the YRD region decreased from 3.44 tons/CNY 10,000 to 1.37 tons/CNY 10,000, an average annual decrease of 4.50%. It shows that the energy utilization rate in the YRD region is increasing year by year, and the dependence of economic development on energy consumption is constantly weakening. However, in 2019–2020, the carbon emission intensity increased significantly from 0.87 tons/CNY 10,000 to 1.37 tons/CNY 10,000, and the increase in carbon emissions in 2019–2020 was small. It can be speculated that the reason for the sharp rise in carbon emission intensity is that the YRD region was affected by the epidemic. Overall, CEECs in the YRD region increased rapidly in the early stage, slowed down and stabilized in the later stage and the carbon emission intensity gradually decreased. The structural improvement of energy was an important factor. That is to say, the proportion of clean energy, such as natural gas, gradually increased, and the proportion of traditional energy, such as gasoline and diesel, gradually decreased.

### 5.2. Spatial and Temporal Distribution Characteristics of Total CEECs

**Figure 4:**
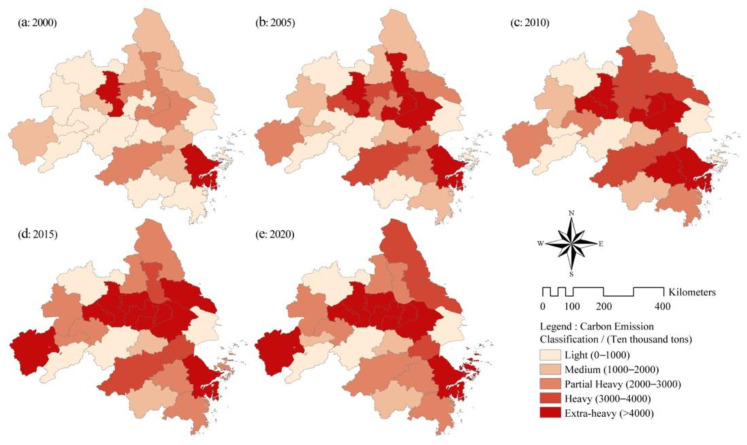
Spatial evolution of carbon emissions of prefecture-level cities in the YRD.

As shown in Figure 4, there were significant spatio-temporal differences in CEECs among the cities in the YRD. Overall, the spatial distribution of carbon emissions was characterized by high levels in the northeast and low levels in the southwest. In 2000, Nanjing and Ningbo had the highest carbon emissions, and the type of CEEC was super heavy (>40 million tons). The CEECs of some cities in the southwestern and southern parts of the YRD region (Chizhou, Xuancheng, Jinhua, etc.) were relatively low, which is related to their low level of economic development and limited industrial technology. In 2005, the carbon emissions of all cities increased to varying degrees, and the number of cities whose carbon emissions were classified as heavy or above increased from two to seven. In particular, the CEECs of some cities in the middle of the YRD region (Nanjing, Wuxi, Suzhou, Taizhou, etc.) increased by more than 5000 × 10^4^ t. The increase in total CEECs in the YRD during this period was related to the fact that cities vigorously developed traditional industries and pursued GDP growth too much, leading to extensive economic development. In 2010, except for in Huzhou, Ningbo, Tongling and Taizhou, carbon emissions decreased; the carbon emissions of other cities still increased to varying degrees, and the number of cities with super-heavy carbon emissions increased from six to seven. In 2015, the CEECs of Hangzhou, Huzhou, Shaoxing, Jinhua, Taizhou, Wuxi, Yangzhou, etc., dropped significantly. The CEECs of other cities began to decline as a whole, and the increase in CEECs narrowed significantly. Nanjing became the only place with an increase in CEECs of more than 5000 × 10^4^ t at city level. This is because, from 2010 to 2015, measures such as low-carbon city construction, low-carbon policies and industrial upgrading in the YRD region were gradually implemented. In 2020, the carbon emissions of Hangzhou, Shaoxing, Jinhua, Ma’anshan, Tongling, Wuxi, Nantong, Taizhou and Shanghai will decrease. However, the overall CEEC increase in the YRD region was about 2053 × 10^4^ t, and showing a rebounding trend. So, from 2015 to 2020, the YRD region needs to pay more attention to reducing emissions of super-heavy cities with carbon emissions (especially in Anqing, Ma’anshan, Changzhou, Wuxi, Zhenjiang, Nanjing and Ningbo).

**Figure 5:**
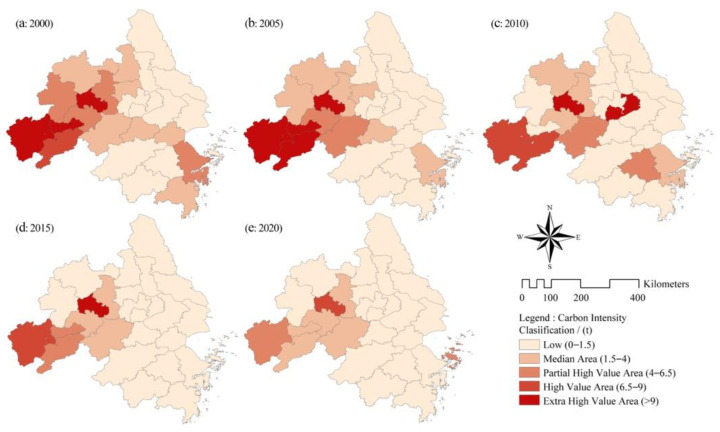
Spatial evolution of carbon intensity of prefecture-level cities in the YRD.

As shown in Figure 5, in 2000, the CEEC intensity of local-level cities was greatest in the low-value area. However, some cities in the southwest (Anqing, Chizhou, Tongling, Ma’anshan) had an intensity mostly higher than 6.5t, because the industry types of these cities mostly consisted of traditional industries, and their economic development depended on high-energy-consuming industries such as petrochemicals, building materials and steel. With time, the high-value areas of carbon emission intensity in the YRD region gradually decreased, and the CEEC intensity of Hefei, Chuzhou, Ningbo and other places dropped significantly, which indicates that the YRD region has taken measures such as industrial supply-side reform and production process improvement in recent years. This promotes the economy of the YRD to move in the direction of low carbonization and high quality. Although carbon emission intensity declined to a certain extent, the trend of a substantial increase in carbon increment and total carbon is not optimistic. It is still necessary to continue to reduce CEEC intensity (especially in Anqing and Ma’anshan) to further reduce the increment of CEECs and control the total amount of CEECs.

### 5.3. The Decoupling between CEECs and Economic Growth

**Figure 6:**
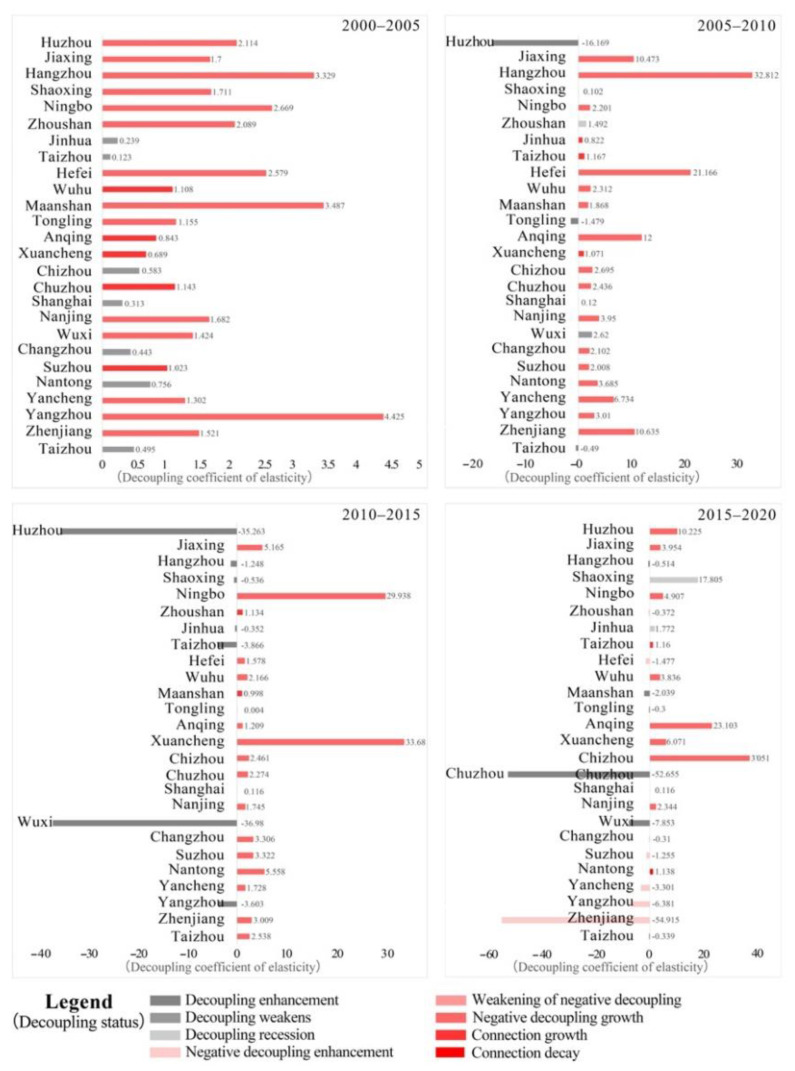
Decoupling of CEEC and industrial output value of prefecture-level cities in the YRD region from 2000 to 2020.

According to the changing characteristics of CEECs in the cities in the YRD region, the changes in CEECs and the gross industrial output value of the cities in the YRD region from 2000 to 2020 were calculated. Moreover, through the Tapio model, the decoupling relationship of the four periods was obtained (Figure 6).

**Figure 7:**
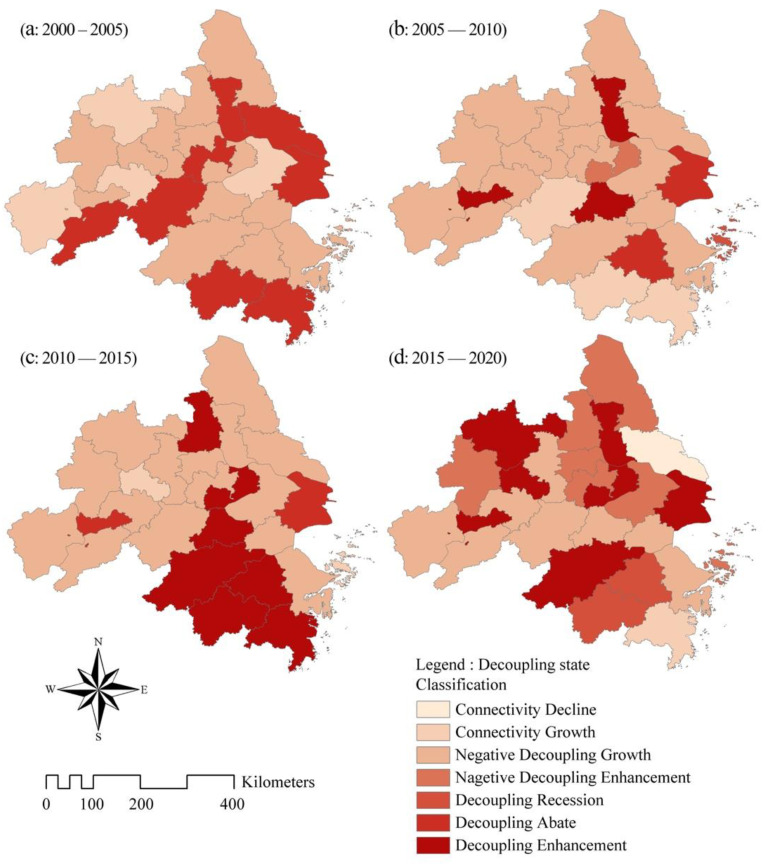
Spatial distribution characteristics of decoupling between CEEC and gross industrial out-put value of prefecture-level cities in the YRD.

From 2000 to 2005, the economic growth and CEECs of most cities in the YRD region were in a state of negative decoupling growth, and the overall decoupling elasticity coefficient was high (Figure 6 and Figure 7). For example, the decoupling elasticity of Yangzhou reached 4.425, which means that the decoupling state was poor. This shows that during this period of economic growth, CEECs were also increasing, but the growth rate of CEECs was much greater than that of economic growth.

From 2005 to 2010, the number of cities in a state of negative decoupling growth continued to increase among local-level cities in the YRD region, reaching 16. However, some cities began to show weakening decoupling and growth in connection, and the decoupling elasticity coefficient decreased significantly. For example, Huzhou changed from the negative decoupling growth state to the decoupling enhanced state, and the decoupling elasticity reached −16.169, which is the most ideal decoupling state. This is because, after the “Eleventh Five-Year Plan” (2001–2005), the YRD region responded to the national call to gradually improve the extensive economic growth model and continuously improve the efficiency of energy utilization. Among the cities, those in the decoupling stage were mainly located in Shanghai, central Zhejiang and central Jiangsu, and those in the negative decoupling stage were mainly located in Anhui, Jiangsu and northern Zhejiang.

From 2010 to 2015, which was China’s “Twelfth Five-Year Plan”, China introduced a large number of emission reduction measures and eliminated outdated production capacity. The total carbon emissions in the YRD region showed a stagnant trend (Figure 2), and many cities showed a state of increasing decoupling. For example, Wuxi actively developed zero-carbon technology, and its decoupling state changed from the negative decoupling growth state in 2000–2005 to the decoupling enhancement state. The decoupling elasticity reached −36.98, which is the most ideal decoupling state. Therefore, the economic growth of the YRD region and CEECs continued to show an increase in decoupling in the long run or a decoupling state alternating between increased decoupling and negative decoupling growth. Among the cities, those in the decoupling stage were mainly located in Shanghai, Shaoxing, Hangzhou, Taizhou, etc. (southern Zhejiang Province, central Zhejiang Province and northern Anhui Province), and those in the negative decoupling stage were mainly located in Wuhu, Anqing, Hefei, Nanjing, etc. (Anhui Province and eastern Jiangsu Province).

From 2015 to 2020, the decoupling index showed large fluctuations. The decoupling relationship between CEECs and economic development in many cities (e.g., Shanghai, Hangzhou, Wuxi, etc.) was further improved, and the decoupling elasticity index continued to decline. However, there were still cities with a high proportion of secondary industries (e.g., Zhenjiang, Anqing, Chizhou) where the decoupling relationship between CEECs and economic development deteriorated to varying degrees. At the same time, the types of decoupling state also increased from three to six, indicating that differences in the level of economic development and economic structure lead to differences in carbon emissions and their decoupling relationship with economic development in different cities in the province.

To sum up, the YRD region has a relatively large proportion of the prefecture-level cities in the state of negative decoupling growth, which shows that an increase in carbon emissions accompanies the economic growth of the YRD region, and the increase in carbon emissions is greater than that of the economy. The rate may be related to the rapid accumulation of cities and towns and the rapid expansion of industries in the YRD. From 2015 to 2020, the number of prefecture-level cities in a state of negative decoupling growth will decrease thanks to the proposal of the “dual-carbon” target in the YRD region and the formulation of related carbon reduction policies. For example, Shanghai proposed to achieve carbon peaking in 2025, Jiangsu Province stated that it would be the first in the county to achieve carbon peaking and the YRD urban agglomeration proposed to achieve carbon peaking in the midterm of the “15th Five-Year Plan”. In the future, the YRD region should further respond to the national call, actively develop the “national low-carbon city pilot”, shut down high-consumption and high-polluting enterprises and carry out industrial upgrading. Through coordinated development, efforts will be made to reverse the relationship between economic growth and CEECs in more cities in the YRD region to decouple growth.

### 5.4. Decomposition Analysis of Influencing Factors of CEEC Decoupling

**Figure 8:**
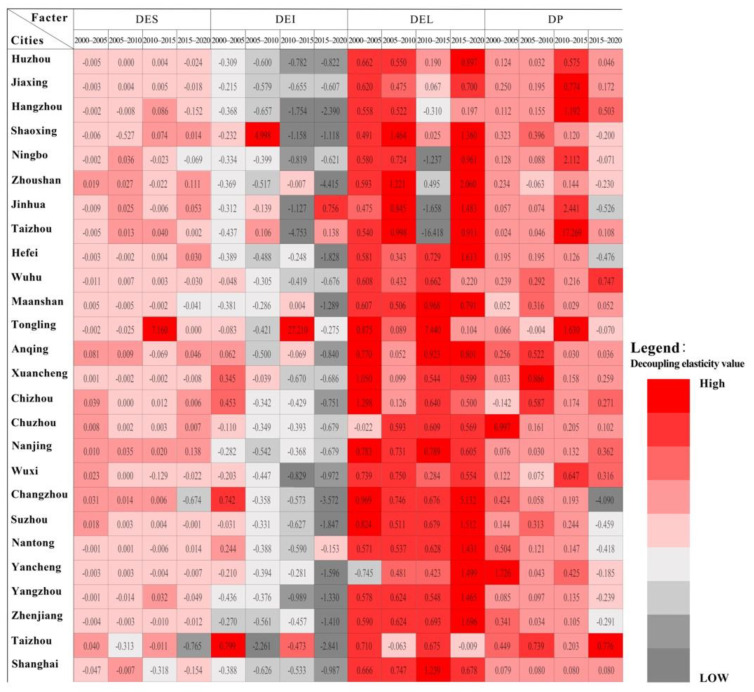
Decomposition of influencing factors of decoupling carbon emissions in prefecture-level cities in the YRD from 2000 to 2020.

Equations (6)–(9) were used to decompose the decoupling elasticity of CEECs in each city in the YRD region to obtain the carbon emissions of four factors: energy structure (*DES*), energy intensity (*DEI*), unit industrial output (*DEL*) and urbanization rate (*DP*). The results are shown in Figure 8.

Overall, from 2000 to 2020, the decoupling elasticity of CEECs for the four factors in the 26 cities was significantly similar (Figure 8). Among them, the decoupling elasticity of *DEL* value was the largest, which was positively correlated with energy carbon emissions as a whole, and its elasticity coefficient was 0.531. The second was the *DP* and *DES*; the impact of *DEI* was the smallest, and its elasticity coefficient was −0.621, decreasing yearly. It can be seen that the “dual-carbon strategy” in the YRD region needs to focus on coordinating the relationship between the quality of industrial production and operation and environmental benefits. In future energy consumption planning, it is necessary to gradually pay attention to the impact of *DP* and *DES* transformation.

#### 5.4.1. The Impact of *DES* on Decoupling of CEECs

From 2000 to 2005, the *DES* of Zhoushan, Ma’anshan, Anqing, Xuancheng, Chizhou, Chuzhou, Nanjing, Wuxi, Changzhou and Suzhou had an impact on economic development. The effect of the carbon emission decoupling relationship was inhibition, and the *DES* of other prefecture-level cities promoted carbon emission decoupling. This shows that from 2000 to 2005, the *DES* of nearly half of the cities in the YRD region did not improve, and the traditional *DES* with high energy consumption was not conducive to local green development.

From 2005 to 2010, for Jiaxing, Ningbo, Jinhua, Taizhou, Wuhu, Anqing, Chuzhou, Nanjing, Changzhou, Suzhou, Nantong and Yancheng, the impact of the *DES* on the decoupling relationship between economic development and carbon emissions was inhibited, and the rest of the prefecture-level cities were promoted. Compared with 2000–2005, the number of cities in which the *DES* inhibited the decoupling of carbon emissions increased by half during this period. This shows that the urban *DES* in the YRD region was not significantly improved at this stage. The traditional *DES* with high energy consumption still inhibited the city’s green development.

From 2010 to 2015, the *DES* of Huzhou, Jiaxing, Hangzhou, Shaoxing, Taizhou, Hefei, Wuhu, Tongling, Chizhou, Chuzhou, Nanjing, Changzhou, Suzhou and Yangzhou had an impact on economic development and carbon emissions. The effect of the decoupling relationship was inhibited, and the remaining prefecture-level cities were promoted. Among them, the absolute value of *DES* in Tongling was the largest, reaching 7.2. During this stage, the number of prefecture-level cities whose *DES* had an inhibitory effect on carbon emission decoupling increased significantly.

From 2015 to 2020, for Taizhou, Tongling, Anqing, Chizhou, Chuzhou, Nanjing, Changzhou, Suzhou, Yancheng, Yangzhou and Zhenjiang, the impact from the DES on the decoupling relationship was inhibition, and the rest of the prefecture-level cities were promoted.

In terms of the number of prefecture-level cities, nearly half of the prefecture-level cities in the YRD region (most of which were located in Anhui and Jiangsu provinces) had a *DES* that inhibited their decoupling of carbon emissions. Still, the absolute value of their *DES* was small. That is, the degree of influence of inhibition was small. From 2000 to 2020, the absolute value of *DES* in prefecture-level cities in the YRD was relatively small, reflecting that the *DES* had an excellent potential for accelerating low-carbon green development in the YRD region. The development of new energy and vigorous promotion of clean energy may become a major starting point for the green development of the region.

#### 5.4.2. The Impact of *DEI* on Decoupling of CEECs

*DEI* refers to the ratio of energy consumption to economic output. From 2000 to 2005, except for Anqing, Xuancheng, Chizhou, Changzhou, Nantong and Taizhou, the impact of *DEI* on the decoupling relationship between economic development and CEECs was inhibited, and the remaining prefecture-level cities were all up for promotion. This shows that from 2000 to 2005, the influencing factor for the low-carbon development of most prefecture-level cities in the YRD region was not *DEI*. From 2005 to 2010, except for Shaoxing, Taizhou, Zhoushan and Wuxi, the impact of *DEI* on the decoupling relationship between economic development and CEECs was inhibited, and other prefecture-level cities were promoted. From 2010 to 2015, only Ma’anshan and Tongling had an inhibitory effect on the decoupling relationship between economic development and CEECs, while other prefecture-level cities promoted it. Among them, the absolute value of *DEI* in Tongling City was the largest, as high as 27.21. It can be seen that between 2005 and 2015, the industry in the YRD region was undergoing continuous transformation; energy consumption changed from low-end extensive to green and intensive and energy efficiency was relatively high. From 2015 to 2020, the *DEI* of Shaoxing, Zhoushan, Taizhou, Hefei, Suzhou, Nantong, Yancheng, Yangzhou and Zhenjiang were decoupled from economic development and CEECs. The influence of the relationship was inhibited, and the rest of the prefecture-level cities were promoted. Although the number of prefecture-level cities increased, the degree of inhibition of the *DES* decreased, and energy utilization began to develop into intensive and efficient development.

#### 5.4.3. The Impact of *DEL* Value on CEEC Decoupling

From 2000 to 2005, only Chuzhou and Yancheng contributed to the decoupling relationship between economic development and CEEC, and the rest of the prefecture-level cities were inhibited. Among them, the absolute value of *DEL* in Chizhou was the largest, reaching 1.3. From 2005 to 2010, only Zhoushan, Wuxi and Taizhou’s *DEL* value contributed to the decoupling relationship between economic development and carbon emissions. The prefecture-level cities were all suppressed, and the absolute value of *DEL* in Shaoxing was the largest, as high as 1.5. This shows that from 2000 to 2010, the industry in the YRD region was in an inefficient development period before the transformation; the unit output value was not high, and the industrial development model was not unbranded and clustered.

From 2015 to 2020, the impact of *DEL* value on the decoupling relationship was manifested in the promotion of prefecture-level cities, including Shaoxing, Zhoushan, Jinhua, Hefei, Changzhou, Suzhou, Nantong, Yancheng, Yangzhou, Zhenjiang and Taizhou. Among them, Changzhou had the largest absolute value of *DEL*, reaching 5.1. This shows that industry in the YRD was developing and transforming in the direction of intensification. Among the cities, the northeastern and southern cities of the YRD (mainly located in Jiangsu Province and Zhejiang Province) had a relatively rapid industrial transformation and development process, which had a relatively significant role in promoting low-carbon economic development.

#### 5.4.4. The Impact of *DP* on CEEC Decoupling

The *DP* of most prefecture-level cities had an inhibitory effect on CEECs. From 2000 to 2020, only the *DP* of Chizhou, Tongling and Ningbo contributed to the decoupling relationship between economic development and CEECs, while the rest of the prefecture-level cities were inhibited. It shows that the rapid urbanization process had a certain inhibitory effect on the low-carbon sustainable development of the YRD urban agglomeration. Behind the increasing *DP* was the extensive urbanization development in the form of extension and spread. Transforming the surging urban population into the driving force for the low-carbon development of the city depended on the industrial upgrading and transformation of the city. As one of the regions with the most active economic development in China, the YRD urban agglomeration will be one of the main areas of low-carbon and green development in the future. The focus of future development will be the elimination, transformation, extension and transfer of the original development methods of some extensive cities in order to promote the transformation and upgrading of their own industries.

#### 5.4.5. Drivers of CEECs by Stage

**Figure 9:**
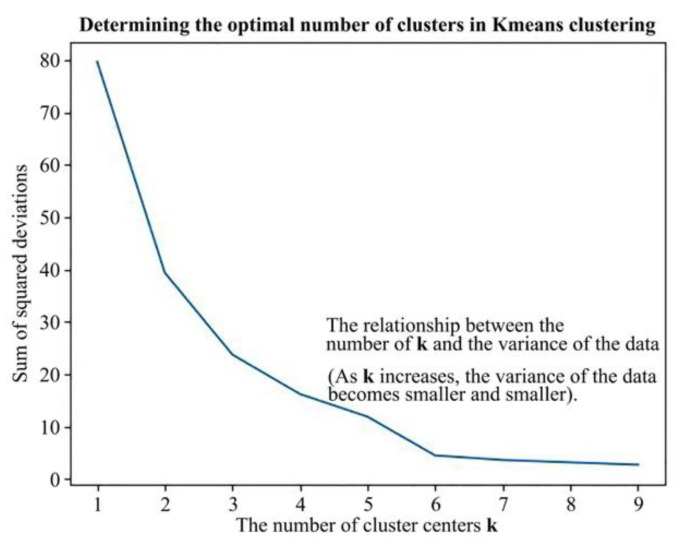
Optimal value of elbow method.

Based on the calculation results of the decoupling elastic coefficients of the four driving factors, the decomposition results of carbon emissions were used as the basis for k-means clustering. The 26 prefecture-level cities were divided into six groups to explore the clustering characteristics of the decoupling elastic coefficients of each city group. The clustering results are shown in Figure 9 and Table 3.

**Figure 10:**
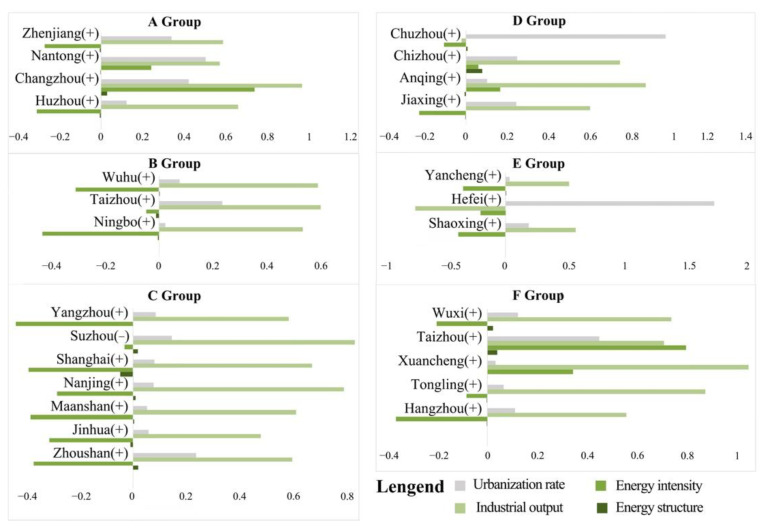
Cluster analysis results in the first period (2000–2005). Note: The symbol (+) after the city indicates an increase in carbon emissions and the symbol (−) indicates a decrease in carbon emissions, the same as in Figure 11, Figure 12 and Figure 13.

The dominant driving factors and driving directions of the carbon emissions of the six groups A–F can be discussed for different periods. The results for the first five years (2000–2005) are shown in Figure 10. During this period, for each group, *DEL* had the largest impact on carbon emissions, followed by *DP* and *DEI*. Among them, *DEL* value and *DP* promoted the growth of carbon emissions, while *DEI* inhibited the growth of carbon emissions. The effect of *DES* was not obvious. It is worth noting that in Group C, the impact of *DEI* on carbon emissions was not negligible and almost corresponded to the impact of *DEL*. In general, because the YRD region paid attention to economic development during this period, secondary industry accounted for a large proportion of the industrial structure, and the energy needed was still dominated by coal. However, the energy utilization efficiency was low, so *DEI* was increasing. The impact of carbon emissions was obvious.

**Figure 11:**
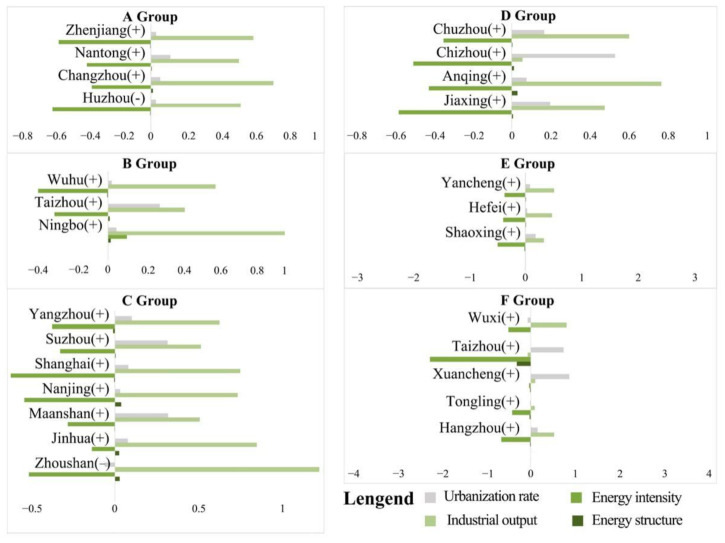
Cluster analysis results in the second five years (2005–2010).

The decomposition results of the second five years (2005–2010) are shown in Figure 11. The main influencing factors in this period were *DEI* and *DEL*. The role of *DEL* was still to promote the growth of carbon emissions, and the role of *DEI* was to suppress the growth of carbon emissions. The obvious difference between this period and the previous period is that the impact of *DEI* greatly increased. In contrast, the impact of *DP* gradually decreased in some groups, especially in groups A, C and D. This characteristic was particularly obvious, which indicates an increased emphasis on energy consumption and environmental protection in the region, and the *DES* of some cities was adjusted during this period. In general, the role of restraining the development of carbon emissions in this period was significantly stronger than that in the first five years, the industrial structure advanced rapidly and the economy developed rapidly. However, the impact of *DEI* on CEECs still needs attention.

**Figure 12:**
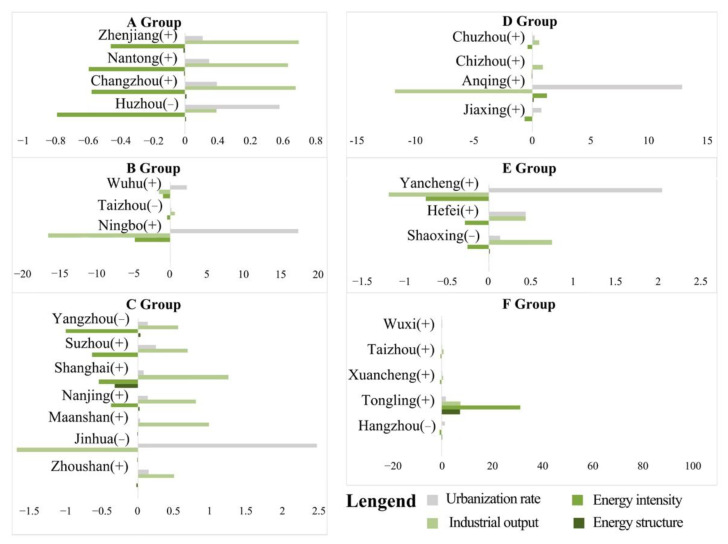
Cluster analysis results in the third five years (2010–2015).

The decomposition results of the third five years are shown in Figure 12. The obvious difference between this period and the previous period is that the effect of suppressing carbon emissions became gradually stronger than that of promotion. *DEI* turned into a major factor contributing to the growth of carbon emissions, especially in groups A, C and E. In addition, the influence of *DP* gradually increased; in groups A and C, *DP* and *DEL* played an essential role in promoting carbon emissions. The biggest feature of Group F was that *DEI* and *DES* had a prominent impact on CEECs, while other influencing factors were not obvious.

**Figure 13:**
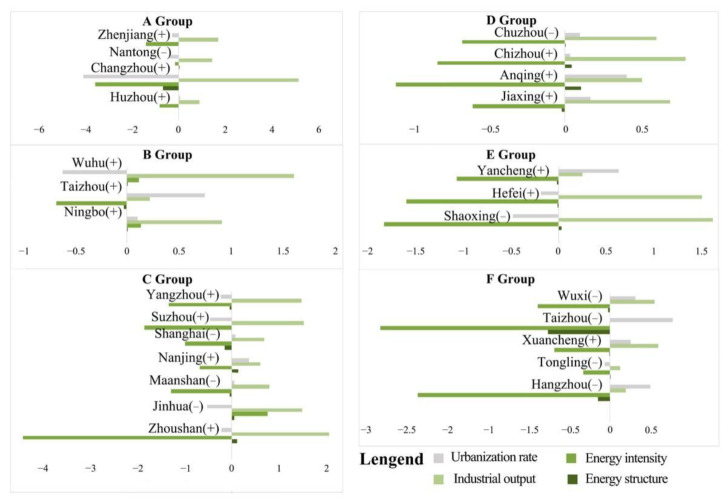
Cluster analysis results in the fourth period (2015–2020).

The decomposition results of the fourth five years are shown in Figure 13. The role of *DEI* in carbon emissions was more critical during this period. The inhibitory effect of this period was similar to that of the previous period and was obviously stronger than the promotion effect. However, compared with the previous groups C, D and E, the intensity of the impact of *DEL* value on promoting carbon emissions increased. In contrast, the impact of *DP* decreased significantly, indicating that the industrial structure of the region still needs to be given attention. It is worth noting that the influence of *DES* increased during this period, especially in group F, which produced a significant inhibitory effect. That shows that the current economic consumption level and population structure changes in some cities had a higher multi-factor impact on carbon emissions than the single impact of population size changes. In general, the total CEECs in some regions declined during this period, but the per capita GDP increased yearly, and the economy developed healthily. However, it is still necessary to pay attention to the efficiency of energy utilization and to strengthen the upgrading of industrial structure and industrial layout to reduce CEECs.

Taken together, the carbon emission patterns and their underlying drivers vary by city group (Table 3). From 2000 to 2015, the correlation between carbon emissions and driving factors was in descending order of *DEL* value, *DP* and *DEI*, and the impact of *DES* could be ignored. The promotion effect of the overall impact factor was greater than the overall inhibitory effect, leading to an overall upward trend in energy carbon emissions in the YRD region in the early stage. However, from 2015 to 2020, the ranking of results was *DEI*, *DEL* value, *DP* and *DES*. The impact of the *DP* dropped significantly, while the impact of the *DES* gradually emerged. The overall inhibitory effect gradually increased and was significantly stronger than the promotion effect, attributed to the optimization of the *DES* and the positive impact of implementing low-carbon policies in recent years.

#### 5.4.6. A Staged Analysis of the Composite Effects of CEEC Drivers

**Figure 14:**
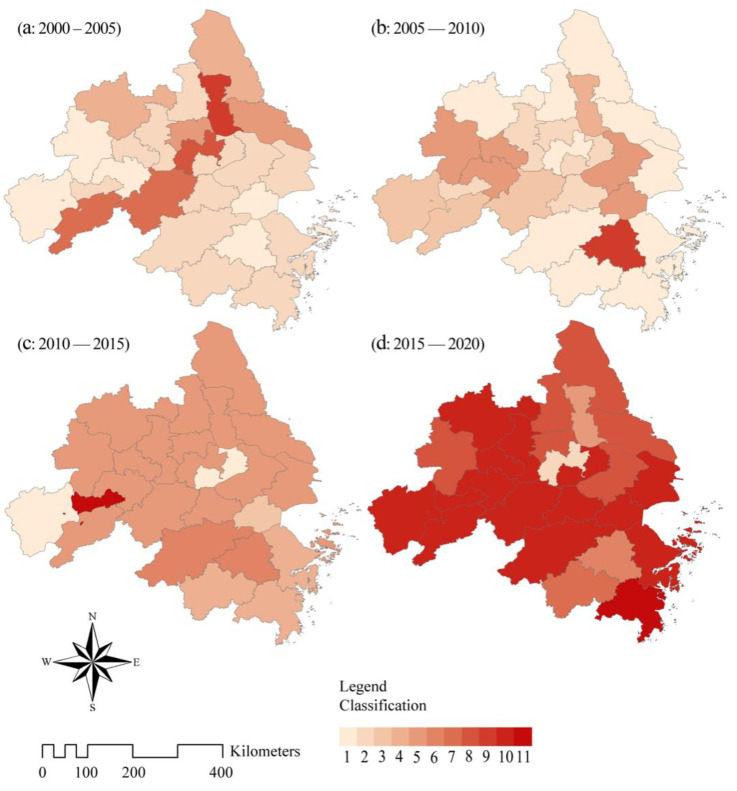
The characteristics of carbon emission composite impact factors in different periods.

The composite effect analysis of the driving factors of CEECs in each period was conducive to revealing the internal mechanism of the temporal and spatial changes of CEECs in cities in the YRD from 2000 to 2020. We numerically numbered the CEECs’ features across the four epochs and reclassified and visualized representations in Arcmap (Table 4 and Figure 14).

In the early stage, cities over-pursued economic development, and the drastic development model brought huge energy consumption. The per *DEL* value became the main and single driving factor in some economically developed cities (mainly concentrated in the more economically developed Zhejiang Province and southern Jiangsu Province and Shanghai: Yangzhou, Nanjing, Wuxi, Suzhou, Shanghai, Ningbo, Huzhou, Hangzhou, Taizhou, and Jinhua). Economically underdeveloped areas (Yancheng, Chuzhou, Tongling, Anqing) were mainly affected by the single factor of *DP*, and other factors were not obvious. At this stage, due to the singleness of the economic development model, about 54% of the cities were affected by the single factor of unit gross industrial output value or *DP*, and only Taizhou and Changzhou were affected by the three factors of unit gross industrial output value, *DP* and *DEI*. In the second and third stages, the influence of the *DP* gradually emerged, and the number of cities affected by the combined influence of the *DEL* value and the *DP* continued to increase. In particular, in the third stage, 17 cities (Hefei, Wuhu, Zhoushan, Yancheng, Chuzhou, Nantong, Zhenjiang, Chizhou, Xuancheng, Taizhou, Changzhou, Yangzhou, Ma’anshan, Nanjing, Suzhou, Shanghai and Huzhou) were affected by the unit industrial total. The dual factors influenced output value and *DP*. At the same time, some cities with prominent tertiary industries (Shaoxing, Hangzhou) were also gradually affected by the impact of the *DES*. At this stage, the number of cities whose carbon emissions were affected by two or three factors increased yearly. In the fourth stage, 15 cities (Anqing, Wuhu, Jiaxing, Zhoushan, Chuzhou, Chizhou, Xuancheng, Ma’anshan, Nanjing, Wuxi, Shanghai, Tongling, Ningbo, Huzhou and Hangzhou) were affected by the unit gross industrial output value, *DP* and the compound influence of the three factors of *DES*, and the four factors of Taizhou were outstanding. At this stage, carbon emissions needed to be controlled by focusing on *DEI* and *DES*.

In general, the carbon emissions of the more developed regions in the early stage were greatly affected by the unit gross industrial output value, and it was necessary to focus on controlling carbon emissions by monitoring the economic development trend. Some economically underdeveloped areas were mainly affected by the *DP*. It should be noted that the *DP* and the huge population base determine that the total carbon emissions of such cities will continue to rise for a long time. In the medium term, affected by the adjustment of economic structure, the cities gradually changed from the single-factor influence in the early stage to the influence of dual influence factors. At this stage, optimizing the industrial structure to control carbon emissions was necessary. In the later stage, the upgrading and transfer of related industries, the transformation of energy consumption forms and the upgrading of industrial layout structure brought diversified development to the city. The amount of carbon emissions tended to be stable, and the impact of a single factor on carbon emissions gradually turned into a composite impact of multiple factors. It can be seen that diversified development of the economy can effectively alleviate the pressure of carbon emissions in many aspects. Therefore, in the process of future economic development, we should pay more attention to the layout of various energy sources and industries, promote the utilization of new energy and renewable energy and promote the development of stagnant industries and the impact of multiple factors, especially *DEI* and *DES*, on carbon emissions so as to explore a reasonable and environmentally friendly carbon emission layout.

## 6. Conclusions and Policy Implications

By 2011, after experiencing a sharp increase in CEECs, the YRD region basically reached a CEECs carbon peak and entered a plateau. Facing the demand for medium and high economic growth in the YRD region in the near future, it will be difficult for the energy consumption and carbon emissions in the YRD region to decrease further. CEECs are expected to decline. Understanding the drivers of CEEC intensity in the 26 prefecture-level cities in the YRD is important for policy making, and decomposition analysis is a useful method for addressing quantitative changes in predetermined benefit factors. This study applied LMDI technology and used an improved Kaya identity to explore the driving factors of CEEC intensity in 26 prefecture-level cities in the YRD from 2000 to 2020. The main conclusions reached are as follows:

MC1: CEECs in the 26 prefecture-level cities in the YRD showed rapid growth in the early stage in the past 20 years, slowed down and stabilized in the later stage and fluctuated during 2009–2011 and 2012–2013. The overall CEEC intensity in the YRD region generally declined. However, there is significant room for reducing CEEC in the YRD. The CEECs in prefecture-level cities showed a more concentrated trend in the central region of the YRD and a trend that increased year by year. The CEEC intensity of the 26 prefecture-level cities was greatest in the low-value area, and the prefecture-level cities with higher intensity were mainly concentrated in the western part of the YRD. The overall carbon emission intensity gradually developed towards the low-value area.

MC2: Among the four driving factors selected and analyzed, the unit industrial output value had the greatest impact on the decoupling relationship between the economic development of prefecture-level cities in the YRD and CEECs, followed by energy intensity. The urbanization rate was the most widely influential factor in the decoupling relationship between economic development and CEECs in the 26 prefecture-level cities. At the same time, the decoupling relationship between CEECs and economic growth in various cities in the YRD region has significant spatial and temporal differences in geographic locations and development stages. In general, the growth rate of CEECs in the YRD region was greater than that of economic growth, which may be related to the rapid expansion of industries in the YRD region. The significant positive interaction between advanced energy structure and economic growth is gradually becoming more prominent.

MC3: The driving factors of the decoupling in the YRD region generally showed a trend changing from unit of gross industrial output value to urbanization rate and energy intensity. This shows that the development and utilization of energy technology and the transformation and upgrading of low-end industries are imminent. The cluster analysis results of the elastic value of driving factors showed that, from 2000 to 2010, the dominant factors affecting the CEECs of the 26 prefecture-level cities were mainly industrial output value and urbanization rate. Overall, the facilitation effect was greater than the inhibitory effect. After 2010, the role of various factors in restraining carbon emissions in the YRD region increased significantly, and the role of energy intensity is still crucial. From 2015 to 2020, the role of energy intensity in CEEC became the dominant factor, and the inhibitory effect gradually became stronger than the promotion effect. In addition, some cities had a relatively good level of urbanization, and the influence of energy structure increased during this period. However, there is still a lot of room for improvement in new energy utilization and low-carbon technology research and development.

MC4: The results of the phase analysis of the composite driving factors of carbon emissions showed that the urban carbon emissions in the YRD region from 2000 to 2010 were mainly affected by a single factor: the economic development area was affected by the industrial output value; the economically underdeveloped area was affected by the urbanization rate impact; carbon emissions showed an upward trend year by year. After 2010, carbon emissions gradually turned into the compound influence of multiple factors, and carbon emissions tended to stabilize. Therefore, in the process of future economic development, encouraging economic diversification, energy structure transformation and model upgrading can effectively alleviate the pressure of carbon emissions in many ways.

According to the discussion above, the YRD region should determine the path to achieve CEEC reduction goals based on the conditions and advantages of the 26 prefecture-level cities and the leading factors driving CEECs. Below are three main policy recommendations based on the above analysis.

PR1: Prefecture-level cities play an important role in China’s administrative system. From the above analysis and the development of the literature, the regulation of provincial governance units urgently needs to be on the prefecture-level-city level. When formulating planning policies, relevant managers should base them on the prefecture-level-city level or even smaller-scale administrative units, thoroughly consider the differences in governance objects and formulate policies for differentiated carbon emissions or to achieve carbon neutrality goals.

PR2: Local-level cities should formulate specific low-carbon development policies based on the contribution rate of major impact factors to CEEC_S_. In the future, the economy of the YRD region will continue to maintain a rapid growth rate. It is not realistic to reduce carbon emissions by reducing the speed of economic development, and prefecture levels should be able to adopt policies tailored to local conditions to reduce CEECs. For pollution-intensive industrial cities, it is necessary to take the lead in promoting the upgrade of the industrial structure. For prefecture-level cities with advanced technology, it is necessary to increase ecological innovation further. Effective carbon emissions trading markets and energy trading markets need to be established. Compared with the terminal processing of carbon emissions trading, energy trading based on source control is more in line with national requirements. For industries characterized by high energy consumption, high pollution, and high carbon emissions, enterprises need to formulate strict development plans and green models suitable for the three main industries. Enterprises should be encouraged to improve energy utilization efficiency and develop a circular economy with low extraction, high use and low emissions. At the same time, it is necessary to increase capital participation in green investment and promote the use of clean energy, such as hydro, wind, solar and many other new energy, renewable electricity and hybrid energy systems. Cities should implement differentiated carbon emission reduction measures. For high-carbon cities, it is necessary to focus on monitoring high-carbon industries and high-carbon enterprises, guide and encourage enterprises to conduct low-carbon operations and reduce carbon emission costs by participating in urban carbon trading. A low-carbon economy is not about no economy but about developing from a bigger economy to a better economy.

PR3: Local-level cities should use the development of new technologies to establish a low-carbon integrated interconnection and mutual assistance strategy network and platform. The YRD is an energy-deficient area, and, as a whole, it is necessary to speed up the research and development of clean energy. At the same time, in the process of industrial structure optimization and new urbanization, attention should be paid to promoting low-carbon industries and regenerative agricultural economic development models. To deal with the dual-carbon goal, we must establish the concept of urban and rural integration, equally treat and guide citizens to establish a zero-carbon life concept, accelerate the promotion of low-carbon lifestyles through modern media network platforms such as WeChat, Douyin and Kuaishou and create a low-carbon consumption atmosphere. Through the participation of the whole population, we will actively build a zero-waste and low-carbon city. We must vigorously develop the digital economy and platform economy, transfer high-energy-consuming industries in terms of industrial structure transformation, optimize the scientific research resources and foreign trade advantages of the YRD, rely on the scientific research resources and foreign trade advantages of the YRD, focus on developing the digital economy and platform economy and reduce dependence on energy consumption.

Our research deeply revealed the dominant factors, decoupling relationships and spatio-temporal variation law of carbon emissions in 26 prefecture-level cities in the YRD from 2000 to 2020. However, the research in this paper still had some limitations. As mentioned above, the influencing factors we chose mainly focused on the industrial development and urbanization of prefecture-level cities, which may not have been comprehensive, and the impact of technological innovation and policies was not quantitatively analyzed. In addition, due to the limitations of data acquisition, it is currently impossible to analyze the spatio-temporal differences within each prefecture-level city in more detail from the perspective of smaller, county-level cities.

## Figures and Tables

**Table 1:** Calculation parameters of carbon emissions.

Energy Types	Standard Coal Coefficient/(kgce/kg)	Carbon Emission Coefficient/(kg/kgce)
Raw coal (tons)	0.7143	0.7559
Washed coal (tons)	0.9	0.7559
Other washed coal (tons)	0.2857	0.7559
Coal products (tons)	0.2857	0.7559
Coke (tons)	0.9714	0.855
Other coking products (tons)	0.9714	0.855
Coke oven gas (10,000 cubic meters)	0.6	0.3548
Blast furnace gas (10,000 cubic meters)	0.1286	0.3548
Converter gas (10,000 cubic meters)	0.2571	0.3548
Producer gas (10,000 cubic meters)	0.1786	0.3548
Natural gas (10,000 cubic meters)	1.2	0.4483
LNG (tons)	1.7572	0.4483
Crude oil (tons)	1.429	0.5857
Gasoline (tons)	1.4714	0.5538
Kerosene (tons)	1.4714	0.5714
Diesel (tons)	1.4571	0.5921
Fuel oil (tons)	1.4286	0.6185
LPG (tons)	1.7143	0.5042
Refinery dry gas (10,000 cubic meters)	1.5714	0.4602
Other petroleum products (tons)	1.7	0.5857
Other fuels (tons)	1	0.7561

**Table 2:** Tapio decoupling system.

Condition		ΔCO_2_/CO_2_	Δ*GIO*/*GIO*	Elasticity (*D*)	Significance
Decoupling entry	Enhance	<0	>0	*D* < 0	In the most ideal state, the carbon emission growth index shows an inverse relationship with economic growth.
	Weaken	>0	>0	0 < *D* < 0.8	The growth rate of carbon emissions is lower than that of economic growth.
	Decline	<0	<0	*D* > 1.2	Carbon emissions decay faster than economic recession.
Negative decoupling	Enhance	>0	<0	*D* < 0	In the most unsatisfactory state, economic growth is negative, and carbon emissions still tend to rise.
	Weaken	<0	<0	0 < *D* < 0.8	Carbon emissions decay faster than economic recession.
	Increase	>0	>0	*D* > 1.2	Carbon emissions are growing faster than economic growth.
Connect	Increase	>0	>0	0.8 < *D* < 1.2	Carbon emissions grow at the same time as the economy, at the same rate and in a linear relationship.
	Decline	<0	<0	0.8 < *D* < 1.2	Carbon emissions and the economy decline at the same time, at the same speed and in a linear relationship.

**Table 3:** Analysis of the main driving factors of each group of urban areas.

	Period							
	2000–2005		2005–2010		2010–2015		2015–2020	
Groups	Main Factor	Average Uncoupling Elasticity	Main Factor	Average Uncoupling Elasticity	Main Factor	Average Uncoupling Elasticity	Main Factor	Average Uncoupling Elasticity
Group A	*DEL*	0.698	*DEL*	0.614	*DEI*	−0.601	*DEL*	2.289
Group B	*DEL*	0.576	*DEL*	0.718	*DP*	6.532	*DEL*	0.697
Group C	*DEL*	0.647	*DEL*	0.741	*DEI*	−0.521	*DEI*	−1.398
Group D	*DEL*	0.667	*DEI*	−0.442	*DEL*	0.56	*DEI*	−0.719
Group E	*DP*	2.243	*DEI*	4.115	*DEI*	−1.687	*DEI*	−4.542
Group F	*DEL*	3.933	*DEI*	−3.824	*DEI*	23.485	*DEI*	−7.164

**Table 4:** Classification of carbon emission characteristics.

Number	Carbon Emission Characteristics
1	*DEL* is the main influencing factor, and the *DP* is outstanding
2	*DEL* is the main influencing factor, and other factors have little influence
3	*DP* is the main influencing factor, and the *DEL* is outstanding
4	*DP* is the main influencing factors, and other factors have little influence
5	*DEL* and *DP* are the main influencing factors, and other factors have little influence
6	*DP* and *DES* are the main influencing factors, and the *DEL* is outstanding
7	*DEL* and *DEI* are the main influencing factors, and other factors have little influence
8	*DEL* and *DES* are the main influencing factors, and the *DP* is outstanding
9	*DEL*, *DP* and *DEI* are the main influencing factors
10	*DEL*, *DP* and *DES* are the main influencing factors
11	*DEL*, *DP*, *DEI* and *DES* are the main influencing factors

## Data Availability

The data are not publicly available due to privacy. The data presented in this paper are available on request from the corresponding author.
